# The Enduring Health Consequences of Combat Trauma: a Legacy of Chronic Disease

**DOI:** 10.1007/s11606-020-06195-1

**Published:** 2020-09-21

**Authors:** Ian J. Stewart, Eduard Poltavskiy, Jeffrey T. Howard, Jud C. Janak, Warren Pettey, Lee Ann Zarzabal, Lauren E. Walker, Carl A. Beyer, Alan Sim, Ying Suo, Andrew Redd, Kevin K. Chung, Adi Gundlapalli

**Affiliations:** 1 Clinical Investigation Facility, David Grant USAF Medical Center, Travis AFB, 101 Bodin Circle, Fairfield, CA 94535 USA; 2 Uniformed Services University of the Health Sciences, Bethesda, MD USA; 3 University of Texas San Antonio, San Antonio, TX USA; 4 Bexar Data, San Antonio, TX USA; 5 VA Salt Lake City Health Care System, Salt Lake City, UT USA; 6 University of Utah School of Medicine, Salt Lake City, UT USA; 7 Defense Health Agency/J6, Randolph AFB, Universal City, TX USA; 8 University of California Davis Medical Center, Sacramento, CA USA

**Keywords:** traumatic injury, cardiovascular disease, diabetes mellitus, veterans health, Military Medicine

## Abstract

**Background:**

A better understanding of the long-term health effects of combat injury is important for the management of veterans’ health in the Department of Defense (DoD) and Veterans Affairs (VA) health care systems and may have implications for primary care management of civilian trauma patients.

**Objective:**

To determine the impact of traumatic injury on the subsequent development of hypertension (HTN), diabetes mellitus (DM), and coronary artery disease (CAD) after adjustment for sociodemographic, health behavior, and mental health factors.

**Design:**

Retrospective cohort study of current and former US military personnel with data obtained from both the DoD and VA health care systems.

**Participants:**

Combat injured (*n* = 8727) service members between 1 February 2002 and 14 June 2016 randomly selected from the DoD Trauma Registry matched 1:1 based on year of birth, sex, and branch of service to subjects that deployed to a combat zone but were not injured.

**Main Measures:**

Traumatic injury, stratified by severity, compared with no documented injury. Diagnoses of HTN, DM, and CAD defined by International Classification of Diseases 9th or 10th Revision Clinical Modification codes.

**Key Results:**

After adjustment, severe traumatic injury was significantly associated with HTN (HR 2.78, 95% CI 2.18–3.55), DM (HR 4.45, 95% CI 2.15–9.18), and CAD (HR 4.87, 95% CI 2.11–11.25), compared with no injury. Less severe injury was associated with HTN (HR 1.14, 95% CI 1.05–1.24) and CAD (HR 1.62, 95% CI 1.11–2.37).

**Conclusions:**

Severe traumatic injury is associated with the subsequent development of HTN, DM, and CAD. These findings have profound implications for the primary care of injured service members in both the DoD/VA health systems and may be applicable to civilian trauma patients as well. Further exploration of pathophysiologic, health behavior, and mental health changes after trauma is warranted to guide future intervention strategies.

**Electronic supplementary material:**

The online version of this article (10.1007/s11606-020-06195-1) contains supplementary material, which is available to authorized users.

## INTRODUCTION

An emerging body of evidence indicates that trauma is associated with chronic medical conditions.^1, 2^ This complements evidence in other populations of patients that suffer acute insults. For example, acute kidney injury has been associated with chronic kidney disease (CKD),^3^ end-stage renal disease,^3^ heart failure,^4, 5^ hypertension (HTN),^1, 6^ and long-term mortality.^7, 8^ Similarly, sepsis in general and pneumonia specifically have been associated with cardiovascular disease, cognitive impairment, and long-term mortality.^9–12^ However, these associations are complicated by the high prevalence of psychiatric morbidity in critically ill patients,^13, 14^ which has in turn been correlated with poor long-term outcomes such as cardiovascular disease.^15^

The primary limitation of the literature so far is selection bias: patients who become acutely ill are fundamentally different from those that do not. From this lens, combat casualties are a particularly interesting group to study. In contrast to other cohorts, there are relatively strict health requirements for both military service and for deployment to a combat theater. Therefore, there is no a priori reason to believe that injured patients are significantly different from non-injured patients at baseline. Furthermore, the continuity of care between the Department of Defense (DoD) and Veterans Affairs (VA) health systems allows for robust follow-up and adjustment for other covariates. Since randomized controlled trials are not possible, retrospective studies from combat casualties are likely to provide the best evidence. Additionally, findings from combat casualties are not only relevant to military service members and veterans but also may elucidate important public health implications for similar civilian trauma such as gun violence.^16, 17^

In the present study, we randomly selected a large cohort of combat casualties and matched them with a population that was deployed to a combat location, but not injured. We hypothesized that combat injured patients would have higher rates of HTN, coronary artery disease (CAD), and diabetes mellitus (DM) compared with uninjured patients after adjustment for demographic, health behavior, and mental health factors.

## METHODS

The study was reviewed and approved by the David Grant USAF Medical Center Institutional Review Board (IRB), the University of Utah IRB, and the Research Review Committee of the VA Salt Lake City Health Care System. We derived two cohorts of military personnel: (1) combat injured and (2) deployed to Iraq or Afghanistan, but not injured. The injured cohort was composed of service members that were wounded in combat operations in Iraq or Afghanistan from 1 February 2002 to 14 June 2016 from the Department of Defense Trauma Registry (DoDTR). A random sample of 10,000 patients was selected from the DoDTR that were injured during the study period. These were matched 1:1 with the control group based on birth year (± 1 year), sex, and branch of service (Army, Air Force, Marines, Navy, and Coast Guard) utilizing the Veterans Affairs/DoD Identity Repository (VADIR). This uninjured group had no documented combat injury in DoDTR and did not have a combat injury separation from service. We opted not to include a group of military members that were not deployed because non-deployed groups have been shown to have higher rates of chronic disease,^18^ likely because deployments are deferred for medical reasons. Additional data were queried from multiple data systems, including the Military Health System Data Repository (MDR), the Joint VA-DoD Suicide Data Repository (SDR),^19^ the Defense Manpower Data Center (DMDC), and the Veterans Informatics and Computing Infrastructure (VINCI).

Data on birth year, sex, rank, and service branch were derived from DoDTR for injured patients and VADIR for uninjured patients. Given the low number of subjects in the Coast Guard, they were included with the Navy for analysis. Rank was categorized into junior enlisted, senior enlisted, and officers as a proxy for socioeconomic status. Data on race/ethnicity were derived primarily from DMDC. If race/ethnicity was missing from DMDC, it was obtained from VADIR; if missing from VADIR, race/ethnicity was derived from MDR. Data on marital status were obtained from MDR. Injury characteristics for the injured cohort were obtained from DoDTR, including injury type, injury mechanism, and Injury Severity Score (ISS). The ISS is a validated, anatomically based scoring system to quantify injury severity, with scores ranging from 1 to 75.^20^ For the purposes of analysis, injury was considered a categorical variable: not injured for the uninjured cohort, and 1–24 (minor to moderate) or ≥ 25 (severe) for the injured cohort. We obtained mortality data from SDR. Subjects were considered tobacco users if they had any evidence of this health behavior in MDR. Patients were considered to have the outcome of HTN, CAD, or DM if they had an International Classification of Diseases 9th or 10th Revision Clinical Modification (ICD-9/10-CM) diagnosis code(s) for a condition of interest in either MDR or VINCI (Supplemental Table 1). To define the presence of the outcome, we utilized a previously published method.^21, 22^ If the diagnosis occurred in an inpatient setting, they were considered to have the outcome. However, if the diagnosis occurred in an outpatient setting, a second code within the next 2 years was required for confirmation. We also considered the development of other ICD-9/10-CM conditions as time-dependent covariates in our analyses, including adjustment disorders, anxiety disorders, insomnia, post-traumatic stress disorder (PTSD), depression, and alcohol dependence (Supplemental Table 1). A patient was considered to have one of these diagnoses if they had two encounters at least 7 days apart with the appropriate diagnosis code.^23^ Obesity was also considered as a time-varying covariate. A patient was considered obese if they had a diagnosis of overweight, obesity, or morbid obesity in the medical record (Supplemental Table 1).

Patients were excluded from the analysis if they could not be matched, died within 90 days of the index date, did not have an encounter in the study period or after the index date, had a pre-existing health condition (HTN, DM, CAD, or CKD) prior to index date, or had a missing variable of interest. Categorical variables are presented as percentages and compared by chi-squared tests. Continuous variables are presented as medians and interquartile ranges (IQR) based on data distribution and compared using Wilcoxon rank-sum tests. For our primary analyses, we utilized Fine and Gray competing risk models.^24^ The index date was the injury date in the injured cohort. For each non-injured patient, the index date was the injury date for the subject they were matched to in the injured cohort. Patients were followed until they had the outcome of interest, died, were lost to follow up, or 14 June 2016 (whichever came first). Stratified models were run for each outcome of interest. These included univariate models and three nested multivariable models: (1) injury status and demographics (age, race/ethnicity, rank, and marital status), (2) injury status, demographics, and health behavior (alcohol dependence, tobacco use, and obesity), and (3) injury status, demographics, health behavior, and mental health factors (adjustment disorder, anxiety disorder, insomnia, PTSD, and depression). Data are presented graphically utilizing cumulative incidence functions. All statistical analyses were performed in SAS version 9.4 (SAS Institute, Cary, NC).

## RESULTS

Of the original 10,000 patients obtained from DoDTR, 346 could not be linked to administrative records. This left 9654 subjects that were matched 1:1 based on age, sex, and service to uninjured subjects. Of these 9654 pairs, 138 (1.4%) were excluded for death within 90 days of the index date, 64 (0.6%) for no encounter during the study period, 578 (5.9%) for no encounter after index date, 118 (1.2%) for pre-existing conditions, and 29 (0.3%) for a missing variable of interest. The final study population was composed of 8727 subjects in the injured and uninjured cohorts, for a total N of 17,454.

**Figure 1:**
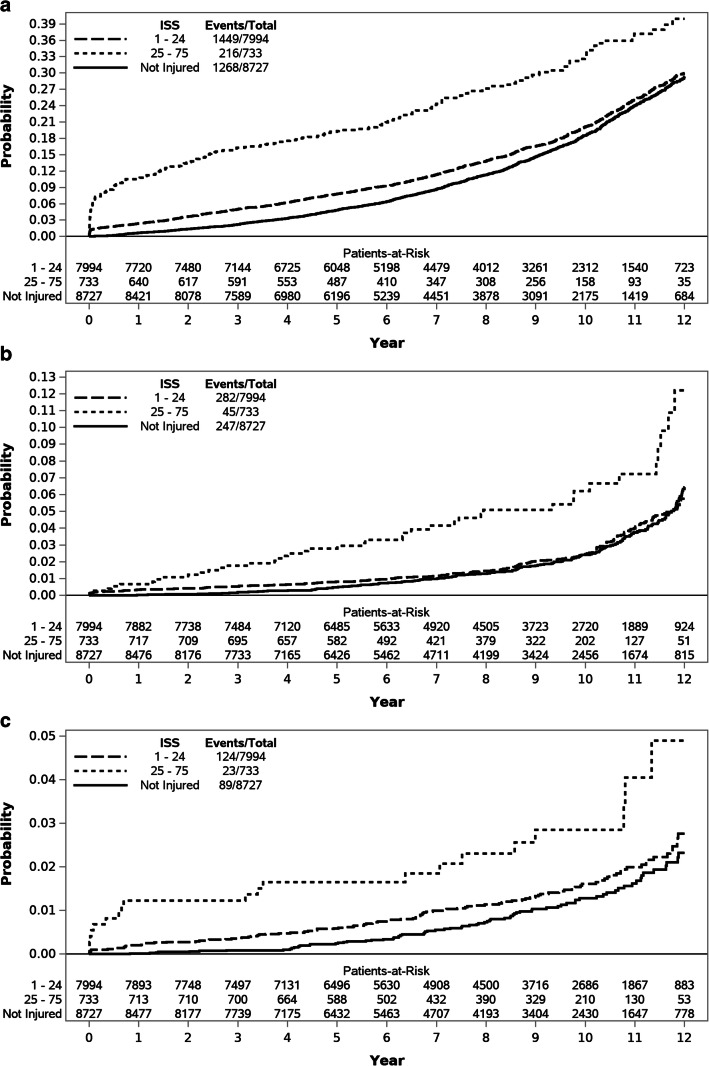
Results for the outcomes of interest. Hypertenstion (panel A), Diabetes Mellitus (panel B), Coronary Artery Disease (panel C).

Characteristics of the study population are presented in Table 1. Patients in the injured cohort were more likely to be married (49.0% vs 46.2%; *p* < 0.001) and less likely to be junior enlisted personnel (58.8% vs 65.7%; *p* < 0.001), compared with the uninjured cohort. Small differences in race/ethnicity were also seen, with injured patients more likely to be non-Hispanic White (75.8% vs 72.0%) and less likely to be non-Hispanic Black (8.5% vs 12.7%). After the index date, injured patients had significantly higher total incidence of HTN (19.4% vs 14.7%; *p ≤* 0.001), DM (3.8% vs 2.9%; *p* < 0.001), and CAD (1.7% vs 1.1%; *p* < 0.001). Injured patients had higher incidence rates per 1000 person years for HTN (17.7 vs 14.2; *p* < 0.001), DM (3.3 vs 2.5; *p* < 0.001), and CAD (1.4 vs 1.1; *p* = 0.003). Injured patients were also more likely to die after 90 days compared with uninjured patients (1.7% vs 1.2%; *p* = 0.015). The median follow-up time was 8.4 years (IQR 5.3–10.6), which differed between groups. Patients in the injured cohort had longer follow-up times (median 8.8, IQR 5.7–10.8) compared with patients in the uninjured cohort (median 7.8, IQR 4.9–10.4; *p* < 0.001). Injured patients had a median ISS of 6 (IQR 2–13) with 8.4% having an ISS ≥ 25 (denoting severe injury). Results for the univariate and fully adjusted multivariable models for the outcomes of interest are presented in Tables 2, 3, and 4. Results of additional nested multivariable modes are presented in Supplemental Tables 2 through 4. Results for the outcomes of interest are displayed graphically, stratified by injury status, in Figure 1.

**Table 1:** Characteristics of the Study Population

	Combined cohort *N* = 17,454	Combat injured *N* = 8727	Deployed, not injured *N* = 8727	*p* value
Age*	24 (22–29)	24 (22–29)	24 (22–29)	0.448
Sex, *N* (%)				1.000
Male	17,112 (98.0)	8556 (98.0)	8556 (98.0)	
Female	342 (2.0)	171 (2.0)	171 (2.0)	
Race/ethnicity, *N* (%)				< 0.001
Non-Hispanic White	12,899 (73.9)	6616 (75.8)	6283 (72.0)	
Hispanic	1838 (10.5)	937 (10.7)	901 (10.3)	
Non-Hispanic Black	1850 (10.6)	742 (8.5)	1108 (12.7)	
Asian^†^	571 (3.3)	284 (3.3)	287 (3.3)	
Other^‡^	296 (1.7)	148 (1.7)	148 (1.7)	
Service, *N* (%)				1.000
Army	12,810 (73.4)	6405 (73.4)	6405 (73.4)	
Air Force	312 (1.8)	156 (1.8)	156 (1.8)	
Marines	3844 (22.0)	1922 (22.0)	1922 (22.0)	
Navy	488 (2.8)	244 (2.8)	244 (2.8)	
Rank, *N* (%)				< 0.001
Junior enlisted	10,861 (62.2)	5127 (58.8)	5734 (65.7)	
Senior enlisted	5350 (30.6)	3028 (34.7)	2322 (26.6)	
Officer	1243 (7.1)	572 (6.6)	671 (7.7)	
Marital status, *N* (%)				< 0.001
Single	9146 (52.4)	4448 (51.0)	4698 (53.8)	
Married	8308 (47.6)	4279 (49.0)	4029 (46.2)	
Hypertension, *N* (%)**	2974 (17.0)	1689 (19.4)	1285 (14.7)	< 0.001
Diabetes, *N* (%)**	578 (3.3)	329 (3.8)	249 (2.9)	< 0.001
Coronary artery disease, *N* (%)**	243 (1.4)	149 (1.7)	94 (1.1)	< 0.001
Death, *N* (%)^††^	250 (1.4)	144 (1.7)	106 (1.2)	0.015
Median follow-up time, years, median (IQR)	8.4 (5.3–10.6)	8.8 (5.7–10.8)	7.8 (4.9–10.4)	< 0.001
Incidence rates per 1000 person years				
Hypertension	15.8	17.7	14.2	< 0.001
Diabetes	2.9	3.3	2.5	< 0.001
Coronary artery disease	1.2	1.4	1.1	0.003

*In years at index date

^†^Including Native Hawaiian and Pacific Islander

^‡^Including multi-racial

**Onset after index date

^††^> 90 days after index date

**Table 2:** Univariate and Final Multivariable Competing Risk Models for the Outcome of Hypertension

	Univariate			Model 3		
	HR	95% CI	*p* value	HR	95% CI	*p* value
Age*	1.08	1.01–1.16	0.037	1.22	1.12–1.32	< 0.001
Race/ethnicity						
NH White	Ref	–	–	Ref	–	–
Hispanic	0.90	0.78–1.05	0.175	0.87	0.73–1.03	0.098
NH Black	1.61	1.42–1.83	< 0.001	1.94	1.69–2.24	< 0.001
Asian	1.21	0.95–1.56	0.130	1.25	0.934–1.66	0.130
Other	1.19	0.85–1.66	0.316	1.18	0.81–1.73	0.385
Rank						
Enlisted (Jr)	Ref	–	–	Ref	–	–
Enlisted (Sr)	1.07	0.95–1.20	0.271	1.00	0.87–1.14	0.982
Officer	0.81	0.68–0.97	0.023	0.98	0.80–1.20	0.851
Married	1.16	1.05–1.28	0.003	1.11	1.00–1.24	0.062
ISS						
Not injured	Ref	–	–	Ref	–	–
1–24	1.26	1.18–1.35	< 0.001	1.14	1.05–1.24	0.002
≥ 25	2.71	2.17–3.40	< 0.001	2.78	2.18–3.55	< 0.001
Alcohol dependence	1.71	1.45–2.01	< 0.001	1.28	1.06–1.54	0.011
Tobacco						
Yes	1.34	1.20–1.50	< 0.001	1.25	1.10–1.43	< 0.001
No	Ref	–	–	Ref	–	–
Unknown	1.14	1.02–1.26	0.020	1.12	0.99–1.26	0.084
Obesity	1.35	1.10–1.65	0.004	1.47	1.15–1.86	0.002
Adjustment disorder	1.54	1.39–1.70	< 0.001	0.98	0.87–1.11	0.771
Anxiety disorder	1.85	1.66–2.05	< 0.001	1.29	1.13–1.48	< 0.001
Insomnia	1.91	1.72–2.11	< 0.001	1.40	1.24–1.59	< 0.001
PTSD	1.84	1.68–2.02	< 0.001	1.24	1.08–1.42	0.002
Depression	1.81	1.64–2.00	< 0.001	1.21	1.05–1.38	0.007

Junior (Jr), senior (Sr), injury severity score (ISS), post-traumatic stress disorder (PTSD), non-Hispanic (NH)

*Per each 1-year increase in age

**Table 3:** Univariate and Final Multivariable Competing Risk Models for the Outcome of Diabetes Mellitus

	Univariate			Model 3		
	HR	95% CI	*p* value	HR	95% CI	*p* value
Age*	0.83	0.68–1.02	0.077	0.90	0.68–1.20	0.475
Race/ethnicity						
NH White	Ref	–	–	Ref	–	–
Hispanic	2.90	1.70–4.93	< 0.001	3.93	1.94–7.95	< 0.001
NH Black	1.36	0.93–1.99	0.109	1.64	1.01–2.66	0.047
Asian	3.10	1.31–7.33	0.010	2.30	0.79–6.72	0.128
Other	5.76	1.67–19.84	0.006	15.44	2.42–98.56	0.004
Rank						
Enlisted (Jr)	Ref	–	–	Ref	–	–
Enlisted (Sr)	0.88	0.62–1.24	0.449	0.87	0.56–1.36	0.542
Officer	0.66	0.36–1.18	0.160	1.13	0.55–2.34	0.738
Married	1.33	0.99–1.78	0.060	1.09	0.74–1.60	0.665
ISS						
Not injured	Ref	–	–	Ref	–	–
1–24	1.22	1.00–1.49	0.048	1.15	0.86–1.53	0.344
≥ 25	4.50	2.14–9.44	< 0.001	4.45	2.15–9.18	< 0.001
Alcohol dependence	1.78	0.98–3.24	0.060	1.33	0.55–3.24	0.531
Tobacco						
Yes	1.33	0.97–1.83	0.077	1.55	0.98–2.46	0.059
No	Ref	–	–	Ref	–	–
Unknown	1.16	0.84–1.60	0.363	1.17	0.75–1.83	0.487
Obesity	1.64	0.95–2.82	0.077	1.54	0.75–3.16	0.244
Adjustment disorder	1.74	1.31–2.33	< 0.001	1.08	0.69–1.69	0.748
Anxiety disorder	1.66	1.22–2.25	0.001	0.66	0.40–1.08	0.099
Insomnia	1.97	1.46–2.66	< 0.001	1.40	0.94–2.08	0.096
PTSD	2.50	1.86–3.37	< 0.001	1.04	0.69–1.56	0.860
Depression	3.58	2.48–5.17	< 0.001	4.01	2.30–6.98	< 0.001

Junior (Jr), senior (Sr), injury severity score (ISS), post-traumatic stress disorder (PTSD), non-Hispanic (NH)

*Per each 1-year increase in age

**Table 4:** Univariate and Final Multivariable Competing Risk Models for the Outcome of Coronary Artery Disease

	Univariate			Model 3		
	HR	95% CI	*p* value	HR	95% CI	*p* value
Age*	1.11	0.86–1.43	0.436	1.57	1.06–2.34	0.025
Race/ethnicity						
NH White	Ref	–	–	Ref	–	–
Hispanic	1.24	0.73–2.13	0.431	0.90	0.50–1.62	0.723
NH Black	1.11	0.74–1.67	0.612	1.15	0.70–1.87	0.584
Asian	0.58	0.10–3.35	0.540	0.43	0.08–2.35	0.329
Other	2.15	0.33–13.93	0.421	1.47	0.20–10.76	0.707
Rank						
Enlisted (Jr)	Ref	–	–	Ref	–	–
Enlisted (Sr)	0.81	0.53–1.25	0.342	0.61	0.34–1.08	0.088
Officer	0.44	0.23–0.82	0.010	0.40	0.17–0.92	0.032
Married	1.06	0.76–1.48	0.736	1.16	0.74–1.80	0.522
ISS						
Not injured	Ref	–	–	Ref	–	–
1–24	1.50	1.18–1.91	0.001	1.62	1.11–2.37	0.013
≥ 25	3.40	1.52–7.59	0.003	4.87	2.11–11.25	< 0.001
Alcohol dependence	2.14	1.09–4.21	0.027	1.07	0.49–2.32	0.867
Tobacco						
Yes	1.26	0.86–1.84	0.229	1.14	0.70–1.84	0.605
No	Ref	–	–	Ref	–	–
Unknown	1.30	0.88–1.93	0.183	1.29	0.75–2.22	0.366
Obesity	0.78	0.39–1.55	0.473	0.91	0.45–1.82	0.790
Adjustment disorder	1.77	1.24–2.52	0.002	1.01	0.61–1.68	0.958
Anxiety disorder	2.05	1.40–2.99	< 0.001	1.21	0.66–2.21	0.531
Insomnia	2.26	1.56–3.28	< 0.001	1.56	0.85–2.88	0.152
PTSD	1.76	1.29–2.40	< 0.001	0.99	0.61–1.62	0.968
Depression	1.92	1.35–2.73	< 0.001	1.27	0.76–2.15	0.363

Junior (Jr), senior (Sr), injury severity score (ISS), post-traumatic stress disorder (PTSD), non-Hispanic (NH)

*Per each 1-year increase in age

Compared with the non-injured cohort (Table 2), severely injured patients had more than two times the unadjusted risk of HTN (hazard ratio (HR) 2.71, 95% confidence interval (CI) 2.17–3.40; *p* < 0.001). Minor to moderately injured patients were also at increased risk in the unadjusted model (HR 1.26, 95% CI 1.18–1.35; *p* < 0.001). The estimated risk (Supplemental Table 2) was not reduced after adjustment for either demographics (model 1) or demographics and health behavior (model 2). However, there was attenuation in the full model that also considered mental health diagnoses for mild to moderately injured patients (HR 1.14, 95% CI 1.05–1.24, *p* = 0.002). The HR for severely injured patients was essentially unchanged (2.78, 95% CI 2.18–3.55; *p* < 0.001). Other statistically significant factors in the fully adjusted model were age (HR 1.22, 95% CI 1.12–1.32; *p* < 0.001), non-Hispanic Black (HR 1.94, 95% CI 1.69–2.24; *p* < 0.001), alcohol dependence (HR 1.28, 95% CI 1.06–1.54; *p* = 0.011), anxiety disorder (HR 1.29, 95% CI 1.13–1.48; *p* < 0.001), insomnia (HR 1.40, 95% CI 1.24–1.59; *p* < 0.001), PTSD (HR 1.24, 95% CI 1.08–1.42; *p* = 0.002), and depression (HR 1.21, 95% CI 1.05–1.38; *p* = 0.007). Tobacco users were also at a risk of HTN after adjustment.

Results for DM are shown in Table 3. The estimated risk for minor to moderate injury was not attenuated until after the addition of mental health diagnoses (HR 1.15, 95% CI 0.86–1.53; *p* = 0.344). Conversely, the estimated risk for more severely injured patients was similar in the univariate (HR 4.50, 95% CI 2.14–9.44; *p* < 0.001) and fully adjusted (HR 4.45, 95% CI 2.15–9.18; *p* < 0.001) models. Other statistically significant variables in the fully adjusted model included Hispanic ethnicity (HR 3.93, 95% CI 1.94–7.95; *p* < 0.001), non-Hispanic Black race/ethnicity (HR 1.64, 95% CI 1.01–2.66; *p* = 0.047), and other race (HR 15.44, 95% CI 2.42–98.56; *p* = 0.004), compared with non-Hispanic Whites. Depression (HR 4.01, 95% CI 2.30–6.98; *p* < 0.001) was also associated with DM after adjustment.

Models for the outcome of CAD are shown in Table 4. In these models, minor to moderate injury was not attenuated in the fully adjusted model (HR 1.62, 95% CI 1.11–2.37; *p* = 0.013) compared with the unadjusted analysis (HR 1.50, 95% CI 1.18–1.91; *p* = 0.001). The estimated risk for more severely injured patients and CAD was also higher in the fully adjusted model (HR 4.87, 95% CI 2.11–11.25; *p* < 0.001) compared with the unadjusted model (HR 3.40, 95% CI 1.52–7.59; *p* = 0.003). Other than injury status, only age (HR 1.57, 95% CI 1.06–2.34; *p* = 0.025) was significantly associated with CAD in the fully adjusted model.

The Martingale-based Supremum test was used to assess the proportional hazards assumption, and injury status did not adhere to the strict assumption of proportionality for each outcome.^25, 26^ The HRs for each outcome are larger earlier and smaller later in the observation period, but strong and significant throughout. After the first year of follow-up, the HRs are essentially proportional, suggesting that non-proportionalities do not constitute meaningful violations that render the results invalid.^26^ Thus, we did not incorporate injury-time interactions in our final models.

## DISCUSSION

We found that traumatically injured patients were more likely to develop HTN, DM, and CAD when compared with uninjured patients. The attributable risk within the injured group ranged from 0.6% for DM to 4.6% for HTN, equating to 55, 80, and 404 excess cases of DM, CAD, and HTN, respectively. Extended to the entire population of battle-injured service members, this would equate to an additional 2628 cases of HTN, 358 cases of DM, and 520 cases of CAD within a relatively young and previously healthy population. Mental health factors were the only variables that meaningfully attenuated the risk from trauma on chronic disease outcomes in the fully adjusted models, predominantly in the minor to moderate injury group.

Prior work by our group in a cohort of critically injured combat casualties demonstrated that increasing ISS was associated with HTN, CAD, DM, and CKD.^1^ Another group has also observed increased risk of cardiovascular disease^27^ and DM^28^ in civilian patients with burn and non-burn trauma. Similar effects have been seen in other critically ill patient populations. One group examining two large cohorts found that patients with pneumonia were more likely to develop cardiovascular disease^9^ and new onset heart failure.^12^ Another study found that sepsis survivors were at an increased risk for both mortality and major adverse cardiovascular events.^10^

A variety of mental health outcomes have been associated with critical illness.^14, 29, 30^ Furthermore, mental health diagnoses have been associated with HTN,^31, 32^ cardiovascular disease,^32^ and DM.^33^ However, ours is the first study to comprehensively examine the magnitude and timing of mental health on subsequent medical comorbidities with long-term follow-up and a non-historical control group. We found that PTSD and anxiety were associated with HTN in the fully adjusted models. Insomnia, a condition associated with both chronic disease and mental health diagnoses,^34^ was associated with HTN after adjustment. Depression was associated with both HTN and DM. Our prior work on this topic has suggested that PTSD and injury severity are independent risk factors for the subsequent development of HTN.^15^ However, the present study suggests that may only be true for more severely injured patients.

Our study also highlights the importance of consolidating DoD and VA data to examine veterans’ health. Utilizing only data from the DoD would have decreased our follow-up time from 8.4 years (IQR 5.3–10.6) to 5.0 years (IQR 2.1–8.9). To our knowledge, only 10 studies have combined data from the DoD and VA data for the purposes of examining veterans’ health.^35–44^ While administrative hurdles exist in combining data from two different federal agencies, the primary issue is one of data integration. Different formats and data dictionaries are compounded by the deep institutional knowledge necessary to properly interpret the individual variables in order to ensure a basic minimum level of semantic interoperability. Future studies will be made much easier by the planned integration of the VA and DoD electronic health records (EHR) systems. However, this will not help those that served prior to the implementation of the common EHR. Therefore, continued efforts to consolidate data between the DoD and VA are warranted. The young men and women that volunteered to serve their country, and in many cases were injured, deserve nothing less.

The implications of these findings may not be limited to the US military. The underlying tenants of our framework might be applicable to civilian trauma. Even if the attributable risk in the civilian population was half of what is estimated in this study, that would imply a meaningful public health burden. Our findings also have important implications for health disparities research given that gun violence disproportionately impacts certain racial/ethnic and socioeconomic groups^45^ which are already known to be at an increased risk of these chronic diseases.^46^

The strengths of our analysis include its large matched cohort design, long follow-up period, and young and healthy population at baseline. However, our work does have some important limitations. First, while our injured and uninjured groups are both unlikely to have significant health problems at baseline, they were not perfectly matched, as evidenced by differences in race/ethnicity, rank, and marital status. While we adjusted for these variables, this variation implies that the injured and uninjured groups might be different in unmeasured ways. For example, we do not have access to measured blood pressure prior to deployment, which could have differed between groups. Second, our analysis is retrospective and relied on ICD-9/10-CM diagnosis codes for both the outcomes and some predictor variables. ICD codes are generally considered to have greater specificity than sensitivity^47^; therefore, we may be missing diagnoses in our cohorts. Third, our data on tobacco use was limited and was unknown for a large proportion of patients (37.5%). Fourth, we do not have data on therapies used to treat injured patients that might have had an impact on the outcomes (e.g., NSAIDs). Fifth, there were differences in follow-up between the injured and non-injured groups. While this is mitigated by our use of a time-to-event analysis and the fact that the uninjured cohort had a long follow-up period, it is important to note that this might result in some bias. Lastly, the results in our cohort of combat casualties may not be generalizable to other populations with higher prevalence of comorbidities at baseline.

We found that combat injury is associated with the subsequent development of HTN, DM, and CAD. These findings are consistent with the hypothesis that the pathophysiologic processes involved in poor long-term outcomes after traumatic injury are a combination of pre-, peri-, and post-injury health promoting and health compromising factors. Many of these health promoting and health compromising factors are modifiable, allowing for targeted interventions aimed at long-term risk reduction and improved quality of life. Since diagnosis, screening, and risk modification will be done in the primary care setting, it is important for clinicians to be aware of the association between injury and chronic disease. This will be especially important as these patients age and are at a greater risk of developing these conditions, particularly DM and CAD, which may take more time to manifest.

## Electronic Supplementary Material

ESM 1 (DOCX 38 kb)

## References

[1] Stewart IJ, Sosnov JA, Howard JT (2015). Retrospective Analysis of Long-Term Outcomes After Combat Injury. Circulation..

[2] **Boos CJ**, **De Villiers N**, **Dyball D**, **McConnell A**, **Bennett AN**. The Relationship between Military Combat and Cardiovascular Risk: A Systematic Review and Meta-Analysis. *Int J Vasc Med* 2019;2019. 10.1155/2019/9849465 10.1155/2019/9849465 PMC6942813 31934451

[3] Coca SG, Singanamala S, Parikh CR (2012). Chronic kidney disease after acute kidney injury: a systematic review and meta-analysis. Kidney Int.

[4] Bansal N, Matheny ME, Greevy RA (2018). Acute Kidney Injury and Risk of Incident Heart Failure Among US Veterans. Am J Kidney Dis.

[5] Go AS, Hsu CY, Yang J (2018). Acute kidney injury and risk of heart failure and atherosclerotic events. Clin J Am Soc Nephrol.

[6] Hsu CY, Hsu RK, Yang J, Ordonez JD, Zheng S, Go AS (2016). Elevated BP after AKI. J Am Soc Nephrol.

[7] Lafrance JP, Miller DR (2010). Acute kidney injury associates with increased long-term mortality. J Am Soc Nephrol.

[8] Sawhney S, Marks A, Fluck N, Levin A, Prescott G, Black C (2017). Intermediate and Long-term Outcomes of Survivors of Acute Kidney Injury Episodes: A Large Population-Based Cohort Study. Am J Kidney Dis.

[9] Corrales-Medina VF, Alvarez KN, Weissfeld LA (2015). Association between hospitalization for pneumonia and subsequent risk of cardiovascular disease. JAMA..

[10] Ou SM, Chu H, Chao PW (2016). Long-Term Mortality and Major Adverse Cardiovascular Events in Sepsis Survivors. A Nationwide Population-based Study. Am J Respir Crit Care Med.

[11] Ehlenbach WJ, Hough CL, Crane PK (2010). Association between acute care and critical illness hospitalization and cognitive function in older adults. JAMA..

[12] Corrales-Medina VF, Taljaard M, Yende S (2015). Intermediate and long-term risk of new-onset heart failure after hospitalization for pneumonia in elderly adults. Am Heart J.

[13] Davydow DS, Katon WJ, Zatzick DF (2009). Psychiatric morbidity and functional impairments in survivors of burns, traumatic injuries, and ICU stays for other critical illnesses: a review of the literature. Int Rev Psychiatry.

[14] Bienvenu OJ, Friedman LA, Colantuoni E (2018). Psychiatric symptoms after acute respiratory distress syndrome: a 5-year longitudinal study. Intensive Care Med.

[15] Howard JT, Sosnov JA, Janak JC (2018). Associations of Initial Injury Severity and Posttraumatic Stress Disorder Diagnoses With Long-Term Hypertension Risk After Combat Injury. Hypertension..

[16] Rowhani-Rahbar A, Zatzick DF, Rivara FP (2019). Long-lasting Consequences of Gun Violence and Mass Shootings. JAMA..

[17] Talley CL, Campbell BT, Jenkins DH (2019). Recommendations from the American College of Surgeons Committee on Trauma’s Firearm Strategy Team (FAST) Workgroup: Chicago Consensus I. J Am Coll Surg.

[18] Granado NS, Smith TC, Swanson GM (2009). Newly reported hypertension after military combat deployment in a large population-based study. Hypertension..

[19] Defense Manpower and Data Center, Sunnyvale, California; Joint Department of Veterans Affairs (VA) and Department of Defense (DoD) Suicide Data Repository - National Death Index (NDI) Extract; http://www.suicideoutreach.org/SDR, Oct 31, 2018.

[20] **Baker SP**, **O’Neill B**, **Haddon W**, **Long WB**. The injury severity score: a method for describing patients with multiple injuries and evaluating emergency care. *J Trauma* 1974;14(3):187-196. http://www.ncbi.nlm.nih.gov/pubmed/4814394. Feb 13, 2020 4814394

[21] Robitaille C, Dai S, Waters C (2012). Diagnosed hypertension in Canada: incidence, prevalence and associated mortality. CMAJ..

[22] **Yoon J**, **Chow A**. Comparing chronic condition rates using ICD-9 and ICD-10 in VA patients FY2014-2016. *BMC Health Serv Res*. 2017;17(1). doi:10.1186/s12913-017-2504-9 10.1186/s12913-017-2504-9 PMC5561575 28818082

[23] Finley EP, Bollinger M, Noël PH (2015). A national cohort study of the association between the polytrauma clinical triad and suicide-related behavior among US Veterans who served in Iraq and Afghanistan. Am J Public Health.

[24] Fine JP, Gray RJ (1999). A Proportional Hazards Model for the Subdistribution of a Competing Risk. J Am Stat Assoc.

[25] Lin DY, Wei LJ, Ying Z (1993). Checking the Cox model with cumulative sums of martingale-based residuals. Biometrika..

[26] **Allison PD**. Survival Analysis Using SAS : A Practical Guide. SAS Institute; 2010.

[27] Duke JM, Randall SM, Fear MW (2017). Long term cardiovascular impacts after burn and non-burn trauma: A comparative population-based study. Burns..

[28] Duke JM, Randall SM, Fear MW, Boyd JH, Rea S, Wood FM (2018). Diabetes mellitus after injury in burn and non-burned patients: A population based retrospective cohort study. Burns..

[29] Van Beusekom I, Bakhshi-Raiez F, Van der Schaaf M, Busschers WB, De Keizer NF, Dongelmans DA (2019). ICU Survivors Have a Substantial Higher Risk of Developing New Chronic Conditions Compared to a Population-Based Control Group. Crit Care Med.

[30] Marra A, Pandharipande PP, Girard TD (2018). Co-Occurrence of Post-Intensive Care Syndrome Problems Among 406 Survivors of Critical Illness. Crit Care Med.

[31] **Kibler JL**, **Joshi K**, **Ma M**. Hypertension in relation to posttraumatic stress disorder and depression in the US National Comorbidity Survey. *Behav Med* 2009;34(4):125-132. 10.3200/BMED.34.4.125-132 10.3200/BMED.34.4.125-132 19064371

[32] Player MS, Peterson LE (2011). Anxiety disorders, hypertension, and cardiovascular risk: a review. Int J Psychiatry Med.

[33] Roberts AL, Agnew-Blais JC, Spiegelman D (2015). Posttraumatic stress disorder and incidence of type 2 diabetes mellitus in a sample of women: A 22-year longitudinal study. JAMA Psychiatry.

[34] Sarsour K, Morin CM, Foley K, Kalsekar A, Walsh JK (2010). Association of insomnia severity and comorbid medical and psychiatric disorders in a health plan-based sample: Insomnia severity and comorbidities. Sleep Med.

[35] Stewart IJ, Sosnov JA, Snow BD (2017). Hypertension after injury among burned combat veterans: A retrospective cohort study. Burns..

[36] **Beyer CA**, **Poltavskiy E**, **Walker LE**, et al. Persistent Opioid Use After Combat Injury and Subsequent Long-term Risk of Abuse. *Ann Surg* November 2019. 10.1097/SLA.0000000000003658 10.1097/SLA.0000000000003658 31714315

[37] Pugh MJ, Swan AA, Carlson KF (2018). Traumatic Brain Injury Severity, Comorbidity, Social Support, Family Functioning, and Community Reintegration Among Veterans of the Afghanistan and Iraq Wars. Arch Phys Med Rehabil.

[38] Swan AA, Amuan ME, Morissette SB (2018). Long-term physical and mental health outcomes associated with traumatic brain injury severity in post-9/11 veterans: A retrospective cohort study. Brain Inj.

[39] Swan AA, Nelson JT, Pogoda TK, Amuan ME, Akin FW, Pugh MJ (2018). Sensory dysfunction and traumatic brain injury severity among deployed post-9/11 veterans: a chronic effects of neurotrauma consortium study. Brain Inj.

[40] Swan AA, Nelson JT, Pogoda TK (2020). Association of Traumatic Brain Injury with Vestibular Dysfunction and Dizziness in Post-9/11 Veterans. J Head Trauma Rehabil.

[41] Copeland LA, Zeber JE, Bingham MO (2011). Transition from military to VHA care: psychiatric health services for Iraq/Afghanistan combat-wounded. J Affect Disord.

[42] **Marceaux JC**, **Soble JR**, **O’Rourke JJF**, et al. Validity of early-onset dementia diagnoses in VA electronic medical record administrative data. *Clin Neuropsychol* October 2019:1-15. 10.1080/13854046.2019.1679889 10.1080/13854046.2019.1679889 PMC13157277 31645200

[43] Nnamani NS, Pugh MJ, Amuan ME (2019). Outcomes of Genitourinary Injury in U.S. Iraq and Afghanistan War Veterans Receiving Care from the Veterans Health Administration. Mil Med.

[44] Pugh MJ, Swan AA, Amuan ME (2019). Deployment, suicide, and overdose among comorbidity phenotypes following mild traumatic brain injury: A retrospective cohort study from the Chronic Effects of Neurotrauma Consortium. PLoS One.

[45] Dare AJ, Irving H, Guerrero-López CM (2019). Geospatial, racial, and educational variation in firearm mortality in the USA, Mexico, Brazil, and Colombia, 1990-2015: a comparative analysis of vital statistics data. Lancet Public Health.

[46] Graham G (2015). Disparities in Cardiovascular Disease Risk in the United States. Curr Cardiol Rev.

[47] Birman-Deych E, Waterman AD, Yan Y, Nilasena DS, Radford MJ, Gage BF (2005). Accuracy of ICD-9-CM codes for identifying cardiovascular and stroke risk factors. Med Care.

