# Supplementary material for: The Enduring Health Consequences of Combat Trauma: a Legacy of Chronic Disease
Source: J Gen Intern Med. 2020 Sep 21;36(3):713–21. doi: 10.1007/s11606-020-06195-1 (PMC7947104; doi:10.1007/s11606-020-06195-1)
Supplement: Supplementary file 1 — (DOCX 38 kb) [file 11606_2020_6195_MOESM1_ESM.docx]

**Supplemental table 1**. International Classification of Diagnoses 9th and 10th revision, clinical modification, codes used for analysis

|  | ICD-9-CM | ICD-10-CM |
| --- | --- | --- |
| Hypertension | '4010'  '4011'  '4019'  '40200'  '40201'  '40210'  '40211'  '40290'  '40291'  '40300'  '40301'  '40310'  '40311'  '40390'  '40391'  '40400'  '40401'  '40402'  '40403'  '40410'  '40411'  '40412'  '40413'  '40490'  '40491'  '40492'  '40493'   \| '40301' \| \| --- \| \| '40310' \| \| '40311' \| \| '40390' \| \| '40391' \| \| '40401' \| \| '40402' \| \| '40403' \| \| '40490' \| \| '40410' \| \| '40411' \| \| '40412' \| \| '40413' \| \| '40400' \| \| '40491' \| \| '40492' \| \| '40493' \| | \| 'I10' \| \| --- \| \| 'I110' \| \| 'I119' \| \| 'I120' \| \| 'I129' \| \| 'I130' \| \| 'I1310' \| \| 'I1311' \| \| 'I132' \| \| 'I120'  'I129'  'I130'  'I1310'  'I1311'  'I132' \| |
| Diabetes | '25000'  '25001'  '25002'  '25003'  '25010'  '25011'  '25012'  '25013'  '25020'  '25021'  '25022'  '25023'  '25031'  '25030'  '25032'  '25033'  '25050'  '25051'  '25052'  '25053'  '25060'  '25062'  '25061'  '25063'  '25070'  '25071'  '25072'  '25073'  '25080'  '25081'  '25082'  '25083'  '25090'  '25091'  '25093'  '25092'  '25040'  '25043'  '25042'  '25041' | \| 'E1010' \| \| --- \| \| 'E1011' \| \| 'E10311' \| \| 'E10319' \| \| 'E10321' \| \| 'E10329' \| \| 'E10331' \| \| 'E10339' \| \| 'E10341' \| \| 'E10349' \| \| 'E10351' \| \| 'E10359' \| \| 'E1036' \| \| 'E1039' \| \| 'E1040' \| \| 'E1041' \| \| 'E1042' \| \| 'E1043' \| \| 'E1044' \| \| 'E1049' \| \| 'E1051' \| \| 'E1052' \| \| 'E1059' \| \| 'E10610' \| \| 'E10618' \| \| 'E10620' \| \| 'E10621' \| \| 'E10622' \| \| 'E10628' \| \| 'E10630' \| \| 'E10638' \| \| 'E10641' \| \| 'E10649' \| \| 'E1065' \| \| 'E1069' \| \| 'E108' \| \| 'E109' \| \| 'E1100' \| \| 'E1101' \| \| 'E11311' \| \| 'E11319' \| \| 'E11321' \| \| 'E11329' \| \| 'E11331' \| \| 'E11339' \| \| 'E11341' \| \| 'E11349' \| \| 'E11351' \| \| 'E11359' \| \| 'E1136' \| \| 'E1139' \| \| 'E1140' \| \| 'E1141' \| \| 'E1142' \| \| 'E1143' \| \| 'E1144' \| \| 'E1149' \| \| 'E1151' \| \| 'E1152' \| \| 'E1159' \| \| 'E11610' \| \| 'E11618' \| \| 'E11620' \| \| 'E11621' \| \| 'E11622' \| \| 'E11628' \| \| 'E11630' \| \| 'E11638' \| \| 'E11641' \| \| 'E11649' \| \| 'E1165' \| \| 'E1169' \| \| 'E118' \| \| 'E119' \| \| '25040' \| \| '25043' \| \| '25042' \| \| '25041' \| \| 'E1021' \| \| 'E1022' \| \| 'E1029' \| \| 'E1121' \| \| 'E1122' \| \| 'E1129' \| \| 'E1321' \| \| 'E1322' \| \| 'E1329' \| \| '25040' \| \| '25043' \| \| '25042' \| \| '25041' \| \| 'E1021' \| \| 'E1022' \| \| 'E1029' \| \| 'E1121' \| \| 'E1122' \| \| 'E1129' \| \| 'E1321' \| \| 'E1322' \| \| 'E1329' \| \| 'E1021'  'E1022'  'E1029'  'E1121'  'E1122'  'E1129'  'E1321'  'E1322'  'E1329' \| |
| Coronary artery disease | '41000'  '41001'  '41002'  '41010'  '41011'  '41012'  '41020'  '41021'  '41022'  '41030'  '41031'  '41032'  '41040'  '41041'  '41042'  '41050'  '41051'  '41052'  '41060'  '41061'  '41062'  '41070'  '41071'  '41072'  '41080'  '41081'  '41082'  '41090'  '41091'  '41092'  '4110'  '4111'  '41181'  '41189'  '412'  '4130'  '4131'  '4139'  '41400'  '41401'  '41402'  '41403'  '41404'  '41405'  '41406'  '41407'  '41410'  '41411'  '41412'  '41419'  '4142'  '4143'  '4144'  '4148'  '4149'  '4292'  '42971'  '42979' | \| 'I200' \| \| --- \| \| 'I201' \| \| 'I208' \| \| 'I209' \| \| 'I2109' \| \| 'I2111' \| \| 'I2119' \| \| 'I2121' \| \| 'I2129' \| \| 'I213' \| \| 'I214' \| \| 'I220' \| \| 'I221' \| \| 'I222' \| \| 'I228' \| \| 'I229' \| \| 'I230' \| \| 'I231' \| \| 'I232' \| \| 'I233' \| \| 'I236' \| \| 'I237' \| \| 'I238' \| \| 'I240' \| \| 'I241' \| \| 'I248' \| \| 'I249' \| \| 'I2510' \| \| 'I25110' \| \| 'I25111' \| \| 'I25118' \| \| 'I25119' \| \| 'I252' \| \| 'I253' \| \| 'I2541' \| \| 'I2542' \| \| 'I255' \| \| 'I256' \| \| 'I25700' \| \| 'I25701' \| \| 'I25708' \| \| 'I25709' \| \| 'I25710' \| \| 'I25711' \| \| 'I25718' \| \| 'I25719' \| \| 'I25720' \| \| 'I25721' \| \| 'I25728' \| \| 'I25729' \| \| 'I25730' \| \| 'I25731' \| \| 'I25738' \| \| 'I25739' \| \| 'I25750' \| \| 'I25751' \| \| 'I25758' \| \| 'I25759' \| \| 'I25760' \| \| 'I25761' \| \| 'I25768' \| \| 'I25769' \| \| 'I25790' \| \| 'I25791' \| \| 'I25798' \| \| 'I25799' \| \| 'I25810' \| \| 'I25811' \| \| 'I25812' \| \| 'I2582' \| \| 'I2583' \| \| 'I2584' \| \| 'I2589' \| \| 'I259' \| \| 'I510' \| |
| Chronic kidney disease | '25040'  '25041'  '25042'  '25043'  '40301'  '40310'  '40311'  '40390'  '40391'  '40400'  '40401'  '40402'  '40403'  '40410'  '40411'  '40412'  '40413'  '40490'  '40491'  '40492'  '40493'  '5851'  '5852'  '5853'  '5854'  '5855'  '5856'  '5859'  '586'  '25040'  '25043'  '25042'  '25041'  '40301'  '40310'  '40311'  '40390'  '40391'  '40401'  '40402'  '40403'  '40490'  '40410'  '40411'  '40412'  '40413'  '40400'  '40491'  '40492'  '40493' | 'E1021'  'E1022'  'E1029'  'E1121'  'E1122'  'E1129'  'E1321'  'E1322'  'E1329'  'I120'  'I129'  'I130'  'I1310'  'I1311'  'I132'  'N181'  'N182'  'N183'  'N184'  'N185'  'N186'  'N189'  'N19'  'V420'  'V560'  'V568'  'Z4822'  'Z4931'  'Z4932'  'Z940'  'E1021'  'E1022'  'E1029'  'E1121'  'E1122'  'E1129'  'E1321'  'E1322'  'E1329'  'I120'  'I129'  'I130'  'I1310'  'I1311'  'I132' |
| Adjustment disorders | \| '3099' \| \| --- \| \| '3090' \| \| '3091' \| \| '30924' \| \| '30928' \| \| '3093' \| \| '3094' \| \| '30929' \| \| '30982' \| \| '30983' \| \| '30989' \| \| '30921' \| \| '30922' \| \| '30923' \| | \| 'F4320' \| \| --- \| \| 'F4321' \| \| 'F4322' \| \| 'F4323' \| \| 'F4324' \| \| 'F4325' \| \| 'F4329' \| \| 'F438' \| \| 'F439' \| |
| Anxiety disorders | \| '30021' \| \| --- \| \| '30022' \| \| '30023' \| \| '30029' \| \| '30020' \| \| '30001' \| \| '30002' \| \| '30009' \| \| '30000' \| | \| 'F4000' \| \| --- \| \| 'F4001' \| \| 'F4002' \| \| 'F4010' \| \| 'F4011' \| \| 'F40210' \| \| 'F40218' \| \| 'F40220' \| \| 'F40228' \| \| 'F40230' \| \| 'F40231' \| \| 'F40232' \| \| 'F40233' \| \| 'F40240' \| \| 'F40241' \| \| 'F40242' \| \| 'F40243' \| \| 'F40248' \| \| 'F40290' \| \| 'F40291' \| \| 'F40298' \| \| 'F408' \| \| 'F409' \| \| 'F410' \| \| 'F411' \| \| 'F413' \| \| 'F418' \| \| 'F419' \| \| 'F422' \| \| 'F423' \| \| 'F424' \| \| 'F428' \| \| 'F429' \| |
| Insomnia | \| '30742' \| \| --- \| \| '30741' \| \| '32702' \| \| '78052' \| \| '32700' \| \| '32701' \| \| '32709' \| | \| 'F510' \| \| --- \| \| 'F5101' \| \| 'F5102' \| \| 'F5103' \| \| 'F5104' \| \| 'F5105' \| \| 'G470' \| \| 'F5109' \| \| 'G4700' \| \| 'G4701' \| \| 'G4709' \| |
| Post-traumatic stress disorder | '30981' | \| 'F4310' \| \| --- \| \| 'F4311' \| \| 'F4312' \| |
| Depression | \| '29621' \| \| --- \| \| '29622' \| \| '29623' \| \| '29624' \| \| '29625' \| \| '29626' \| \| '29620' \| \| '311' \| \| '29631' \| \| '29632' \| \| '29633' \| \| '29634' \| \| '29630' \| \| '29635' \| \| '29636' \| \| '29699' \| \| '29630' \| \| '3004' \| \| '29690' \| | \| 'F320' \| \| --- \| \| 'F321' \| \| 'F322' \| \| 'F323' \| \| 'F324' \| \| 'F325' \| \| 'F3281' \| \| 'F3289' \| \| 'F329' \| \| 'F330' \| \| 'F331' \| \| 'F332' \| \| 'F333' \| \| 'F3340' \| \| 'F3341' \| \| 'F3342' \| \| 'F338' \| \| 'F339' \| \| 'F341' \| \| 'F3481' \| \| 'F3489' \| \| 'F349' \| \| 'F39' \| |
| Alcohol dependence | \| '30390' \| \| --- \| \| '30391' \| \| '30392' \| \| '30393' \| \| '30300' \| \| '30301' \| \| '30302' \| \| '30303' \| \| '2910' \| \| '30300' \| \| '30301' \| \| '30302' \| \| '30303' \| \| '29181' \| \| '2910' \| \| '29181' \| | \| 'F1020' \| \| --- \| \| 'F1021' \| \| 'F10220' \| \| 'F10221' \| \| 'F10229' \| \| 'F10230' \| \| 'F10231' \| \| 'F10232' \| \| 'F10239' \| \| 'F1024' \| \| 'F10250' \| \| 'F10251' \| \| 'F10259' \| \| 'F1026' \| \| 'F1027' \| \| 'F10280' \| \| 'F10281' \| \| 'F10282' \| \| 'F10288' \| \| 'F1029' \| |
| Overweight, Obesity, and Morbid Obesity | \| '27800' \| \| \| \| --- \| --- \| --- \| \| '27803' \| \| \| \| 'E660' \| \| \| \| 'E6609' \| \| \| \| 'E661' \| \| \| \| 'E668' \| \| \| \| 'E669' \| \| \| \| 'V853' \| \| \| \| 'V8530' \| \| \| \| 'V8531' \| \| \| \| 'V8532' \| \| \| \| 'V8533' \| \| \| \| 'V8534' \| \| \| \| 'V8535' \| \| \| \| 'V8536' \| \| \| \| 'V8537' \| \| \| \| 'V8538' \| \| \| \| 'V8539' \| \| \| \| 'E663' \| \| \| \| '27802' \| \| \| \| 'V852' \| \| \| \| 'V8521' \| \| \| \| 'V8522' \| \| \| \| 'V8523' \| \| \| \| 'V8524' \| \| \| \| 'V8525' \| \| \| \| '27801' \| \| \| \| '27803' \| \| \| \| 'V854' \| \| \| \| 'V8541' \| \| \| \| 'V8542' \| \| \| \| 'V8543' \| \| \| \| 'V8544' \| \| \| \| 'V8545' \| \| \| | \| 'Z683' \| \| --- \| \| 'Z6830' \| \| 'Z6831' \| \| 'Z6832' \| \| 'Z6833' \| \| 'Z6834' \| \| 'Z6835' \| \| 'Z6836' \| \| 'Z6837' \| \| 'Z6838' \| \| 'Z6839' \| \| 'Z6825' \| \| 'Z6826' \| \| 'Z6827' \| \| 'Z6828' \| \| 'Z6829' \| \| 'E662' \| \| 'Z684' \| \| 'Z6841' \| \| 'Z6842' \| \| 'Z6843' \| \| 'Z6844' \| \| 'Z6845' \| \| 'E6601' \| |

Supplemental Table 2. Competing risk models for the outcome of hypertension

|  | Model 1 | | | Model 2 | | |
| --- | --- | --- | --- | --- | --- | --- |
|  | HR | 95% CI | P value | HR | 95% CI | P value |
| Age* | 1.22 | 1.13-1.32 | <0.001 | 1.23 | 1.14-1.34 | <0.001 |
| Race/Ethnicity | | | | | | |
| NH White | Ref | - | - | Ref | - | - |
| Hispanic | 0.85 | 0.73-0.99 | 0.038 | 0.86 | 0.73-1.01 | 0.062 |
| NH Black | 1.79 | 1.57-2.05 | <0.001 | 1.86 | 1.62-2.14 | <0.001 |
| Asian | 1.17 | 0.90-1.53 | 0.245 | 1.17 | 0.89-1.54 | 0.270 |
| Other | 1.20 | 0.84-1.70 | 0.312 | 1.12 | 0.79-1.59 | 0.529 |
| Rank | | | | | | |
| Enlisted (Jr) | Ref | - | - | Ref | - | - |
| Enlisted (Sr) | 0.94 | 0.83-1.06 | 0.292 | 0.96 | 0.85-1.09 | 0.519 |
| Officer | 0.78 | 0.65-0.95 | 0.013 | 0.83 | 0.68-1.01 | 0.061 |
| Married | 1.17 | 1.05-1.30 | 0.003 | 1.16 | 1.04-1.29 | 0.006 |
| ISS | | | | | | |
| Not injured | Ref | - | - | Ref | - | - |
| 1-24 | 1.37 | 1.27-1.47 | <0.001 | 1.37 | 1.28-1.48 | <0.001 |
| ≥25 | 3.13 | 2.48-3.96 | <0.001 | 3.18 | 2.49-4.04 | <0.001 |
| Alcohol Dependence |  |  |  | 1.69 | 1.42-2.01 | <0.001 |
| Tobacco | | | | | | |
| Yes |  |  |  | 1.32 | 1.16-1.49 | <0.001 |
| No |  |  |  | Ref | - | - |
| Unknown |  |  |  | 1.14 | 1.01-1.28 | 0.035 |
| Obesity |  |  |  | 1.57 | 1.26-1.97 | <0.001 |

Abbreviations: junior (Jr), senior (Sr), injury severity score (ISS), non-Hispanic (NH)

*Per each 1 year increase in age

Supplemental Table 3. Competing risk models for the outcome of diabetes mellitus

|  | Model 1 | | | Model 2 | | |
| --- | --- | --- | --- | --- | --- | --- |
|  | HR | 95% CI | P value | HR | 95% CI | P value |
| Age* | 0.85 | 0.65-1.10 | 0.217 | 0.88 | 0.67-1.16 | 0.363 |
| Race/Ethnicity | | | | | | |
| NH White | Ref | - | - | Ref | - | - |
| Hispanic | 3.58 | 1.96-6.55 | <0.001 | 3.77 | 2.05-6.93 | <0.001 |
| NH Black | 1.70 | 1.14-2.53 | 0.009 | 1.65 | 1.10-2.48 | 0.015 |
| Asian | 3.35 | 1.38-8.14 | 0.007 | 2.96 | 1.15-7.64 | 0.025 |
| Other | 8.43 | 1.91-37.24 | 0.005 | 9.53 | 1.42-63.99 | 0.020 |
| Rank | | | | | | |
| Enlisted (Jr) | Ref | - | - | Ref | - | - |
| Enlisted (Sr) | 0.73 | 0.49-1.09 | 0.121 | 0.74 | 0.50-1.11 | 0.151 |
| Officer | 0.68 | 0.36-1.29 | 0.238 | 0.72 | 0.37-1.40 | 0.332 |
| Married | 1.20 | 0.87-1.66 | 0.278 | 1.21 | 0.86-1.70 | 0.270 |
| ISS | | | | | | |
| Not injured | Ref | - | - | Ref | - | - |
| 1-24 | 1.37 | 1.09-1.74 | 0.008 | 1.43 | 1.11-1.83 | 0.005 |
| ≥25 | 5.02 | 2.16-11.67 | <0.001 | 4.96 | 2.20-11.18 | <0.001 |
| Alcohol Dependence |  |  |  | 2.20 | 0.90-5.36 | 0.083 |
| Tobacco | | | | | | |
| Yes |  |  |  | 1.29 | 0.89-1.86 | 0.174 |
| No |  |  |  | Ref | - | - |
| Unknown |  |  |  | 1.11 | 0.74-1.65 | 0.619 |
| Obesity |  |  |  | 1.70 | 0.87-3.32 | 0.121 |

Abbreviations: junior (Jr), senior (Sr), injury severity score (ISS), non-Hispanic (NH)

*Per each 1 year increase in age

Supplemental Table 4. Competing risk models for the outcome of coronary artery disease

|  | Model 1 | | | Model 2 | | |
| --- | --- | --- | --- | --- | --- | --- |
|  | HR | 95% CI | P value | HR | 95% CI | P value |
| Age* | 1.67 | 1.14-2.45 | 0.008 | 1.65 | 1.12-2.43 | 0.011 |
| Race/Ethnicity | | | | | | |
| NH White | Ref | - | - | Ref | - | - |
| Hispanic | 1.03 | 0.57-1.85 | 0.921 | 1.06 | 0.60-1.86 | 0.849 |
| NH Black | 1.22 | 0.80-1.86 | 0.362 | 1.26 | 0.80-1.98 | 0.317 |
| Asian | 0.38 | 0.06-2.58 | 0.321 | 0.40 | 0.06-2.53 | 0.327 |
| Other | 1.33 | 0.21-8.20 | 0.762 | 1.37 | 0.21-8.82 | 0.740 |
| Rank | | | | | | |
| Enlisted (Jr) | Ref | - | - | Ref | - | - |
| Enlisted (Sr) | 0.60 | 0.33-1.08 | 0.087 | 0.59 | 0.33-1.05 | 0.071 |
| Officer | 0.33 | 0.15-0.74 | 0.007 | 0.35 | 0.15-0.78 | 0.011 |
| Married | 1.12 | 0.76-1.67 | 0.566 | 1.12 | 0.75-1.68 | 0.574 |
| ISS | | | | | | |
| Not injured | Ref | - | - | Ref | - | - |
| 1-24 | 1.97 | 1.40-2.79 | <0.001 | 1.93 | 1.36-2.74 | <0.001 |
| ≥25 | 4.46 | 1.95-10.24 | <0.001 | 4.46 | 1.99-9.96 | <0.001 |
| Alcohol Dependence |  |  |  | 1.59 | 0.78-3.26 | 0.201 |
| Tobacco | | | | | | |
| Yes |  |  |  | 1.27 | 0.83-1.94 | 0.267 |
| No |  |  |  | Ref | - | - |
| Unknown |  |  |  | 1.32 | 0.77-2.27 | 0.305 |
| Obesity |  |  |  | 1.08 | 0.53-2.19 | 0.833 |

Abbreviations: junior (Jr), senior (Sr), injury severity score (ISS), non-Hispanic (NH)

*Per each 1 year increase in age
